# FGF21 functions as a sensitive biomarker of APAP-treated patients and mice

**DOI:** 10.18632/oncotarget.17966

**Published:** 2017-05-18

**Authors:** Rong Li, Chao Guo, Xinmou Wu, Zhaoquan Huang, Jian Chen

**Affiliations:** ^1^ Key Laboratory of Tumor Immunology and Microenvironmental Regulation, Guilin Medical University, Guangxi, Guilin 541004, PR China; ^2^ Department of Pharmacy, Guigang City People's Hospital, The Eighth Affiliated Hospital of Guangxi Medical University, Guigang, Guangxi 537100, PR China; ^3^ Department of Pathology, Affiliated Hospital of Guilin Medical University, Guangxi, Guilin 541004, PR China; ^4^ Department of Pharmacy, Guangxi Medical University, Guangxi, Nanning 530021, PR China

**Keywords:** acetaminophen, liver impairment, biomarker, FGF21

## Abstract

Acetaminophen (APAP) is a common medication that induces hepatocellular damage in a time- or dose-dependent manner. Fibroblast growth factor 21 (FGF21) exerts a series of biological effects, including cellular repair. Compared to clinical diagnosis parameters, we aimed to evaluate whether FGF21 can serve as a sensitive biomarker for APAP-induced liver impairment. In the present study, we discussed comparable data from APAP-treated patients and parallelly established APAP-exposed mice for investigation. The resulting human serological data showed that APAP-treated patients have a visible reduction of FGF21 expression in undetected liver impairment of clinical diagnosis. In the animal study, APAP-exposed livers exhibited normal metabolic functions and liver functions, as revealed by biochemical test and histopathological examination. Endogenous FGF21 concentrations in APAP-treated mice were decreased in sera and liver cells. Moreover, comparable immunoassay data showed that hepatocellular FGF21 expression was reduced in a time-dependent manner. Taken together, these findings elucidate the involvement of abnormal FGF21 expression in early APAP-induced liver impairment. Interestingly, FGF21 may be a promising biomarker of APAP-exposed livers.

## INTRODUCTION

Liver injury represents a series of trauma sustained to the liver, characterized by hepatocellular necrosis and inflammation [1]. Medication has been found to induce approximately 70% of drug-mediated liver damage. Acetaminophen (APAP) is a widely used antipyretic and analgesic drug [2] with high effectiveness, potentially generating dose-dependent side-effect over time, such as gastrointestinal irritation and liver injury [3]. APAP has been reported to cause hepatotoxicity that results in acute liver failure in community cases. Overdose of APAP in adults is defined as taking up to 4 g/day regularly [4]. Toxicologically, the culprit of hepatotoxicity induced by APAP is linked to its quinone metabolite, a highly toxic substance that damages liver cells [5]. Because the recommended dose of APAP appears to be safe, it remains a first-line drug for pain and fever management [6]. Despite potential risks, APAP-induced immediate impact on liver function prior to detectable liver impairment has not yet investigated. Fibroblast growth factor 21 (FGF-21) exerts wide mitogenic and cell survival actions involving in morphogenesis, cell growth, embryonic development, and tissue repair [7–8], implying that it may play a role in the pathogenesis of early liver damage. In the current study, our aims were targeted to pool and dissect comparable findings of clinical and rodent experimentations, and to further discuss the possible mechanism of FGF21-impaired liver biofunction induced by APAP.

## RESULTS

### Characterization of diagnostic biomarkers in APAP-treated patients

In instrumental assay using HPLC, the detectable levels of APAP-treated patients were 13.23 ± 4.08 and 9.15 ± 2.76 μg/ml during 1 and 2-h time-points, in which pharmacokinetic profiles showed medicative concentrations. As revealed in Table 1, APAP-administered patients exhibited no significant statistics of clinical biomarkers in liver functions (*P* > 0.05). More interestingly, higher levels of plasma FGF21 were found in APAP-treated subjects than in APAP-free controls (*P* < 0.05) at different time-points. As a result, the comparable data of FGF21 indicated that its sensitivity was more significant than other referenced parameters in APAP-affected liver functions.

**Table 1:** The clinically diagnostic profiles of APAP-treated patients

Biomarkers	Ctrl (1 h)	APAP (1 h)	Ctrl (2 h)	APAP (2 h)
TBIL(U/L)	7.23 ± 1.62	11.03 ± 2.62	6.73 ± 1.57	10.57 ± 1.76
DBIL (μmol/L)	2.83 ± 0.85	3.67 ± 0.95	2.73 ± 0.42	3.13 ± 0.91
ALT(U/L)	18.33 ± 2.52	17.67 ± 5.03	16.00 ± 2.00	16.33 ± 4.16
AST(U/L)	22.00 ± 3.61	20.67 ± 4.16	23.00 ± 3.46	23.00 ± 4.36
ALP (U/L)	78.00 ± 8.89	73.67 ± 9.71	81.67 ± 16.80	75.67 ± 18.15
GGT (U/L)	13.00 ± 2.65	15.67 ± 3.06	25.33 ± .53	23.33 ± 2.08
TBA (μmol/L)	24.17 ± 8.99	15.73 ± 5.05	11.77 ± 2.60	10.47 ± 2.21
5-NT (U/L)	3.90 ± 1.08	3.67 ± 1.03	4.63 ± 1.27	4.70 ± 1.48
TP (g/L)	72.60 ± 1.91	67.30 ± 6.93	79.90 ± 3.63	74.60 ± 4.20
ALB (g/L)	51.30 ± 1.83	49.07 ± 5.36	55.03 ± 1.17	53.07 ± 4.64
Blood-APAP (μg/ml)	-	13.23 ± 4.08	-	9.15 ± 2.76
Serum-FGF21 (nmol/mL)	6.46 ± 0.99	0.28 ± 0.07^a^	6.61 ± 0.94	0.24 ± 0.05^a^

Note:Ctrl, control; APAP, acetaminophen; TBIL, total bilirubin; DBIL, direct bilirubin; ALT, glutamic-pyruvic transaminase; AST, glutamic-oxaloacetic transaminase; ALP, alkaline phosphatase; GGT, glutathione transpeptidase; TBA total, bile acids; 5-NT, 5-nucleotide enzyme; TP, total protein; ALB, albumin; hFGF21, human fibroblast growth factor 21.

^a^*P* < 0.05 vs Ctrl statistically analyzed through Student's *t* test.

### The impact of APAP on metabolic parameters in mice

**Figure 1:**
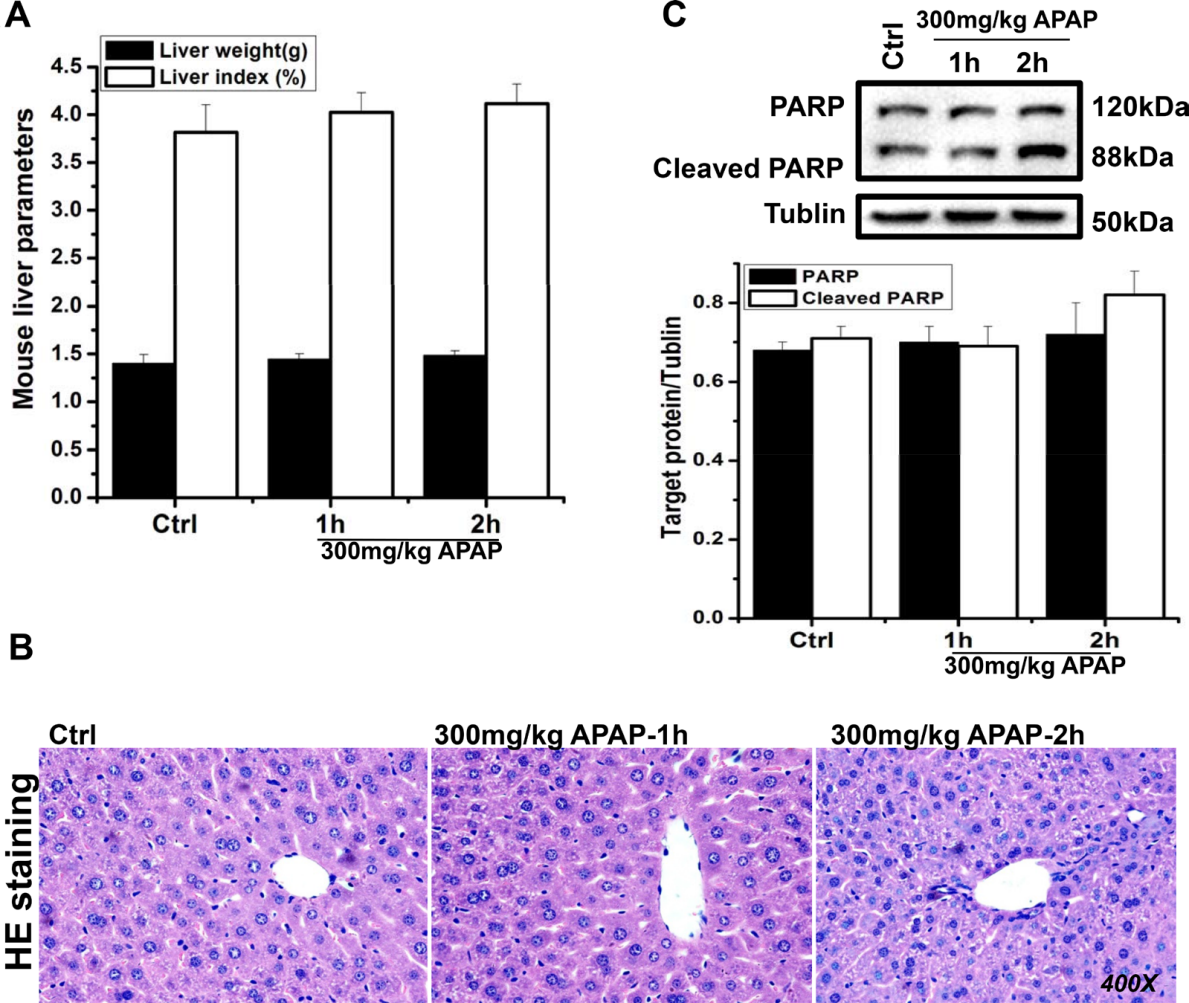
Biological characterization of the effect of APAP on mouse liver cell functions The recorded data exhibited no significant difference in liver mass of APAP-treated and APAP-free mice (**A**). HE staining (magnification 400×) revealed no liver impairment in APAP-exposed livers (**B**). Western blotting data showed no statistically significant difference in early apoptosis-related PARP and cleaved-PARP expression in all liver samples (**C**).

To evaluate the effect of APAP on metabolic functions, liver injury-free mice treated with APAP were established. Basally, APAP-treated mice showed no comparable data of liver weight and liver index when compared to that in mice from the control group (*P* > 0.05; Figure 1A). In morphological observation (HE staining), both APAP-exposed and APAP-free livers had normal cytoarchitecture and hepatocellular numbers, characterized with no visible signs of liver impairment (Figure 1B). In addition, western blotting data found that PARP and its cleaved phenotype (early apoptosis-screened indicator) expression in both APAP-treated and APAP-free livers showed no statistical significance (*P* > 0.05; Figure 1C). As shown in Table 2, basal metabolic parameters (such as glucose, insulin, glucagon, blood lipids) and liver functional enzymes (alanine aminotransferase [ALT] and aspartate aminotransferase [AST]) in these livers exhibited no significant differences (*P* > 0.05). In comparison to APAP-free liver, serum and liver FGF21 levels in APAP-exposed mice showed increased expression, especially during the 2-h exposure (*P* < 0.05).

**Table 2:** The characterized parameter in APAP-treated mice

Biomarkers	Ctrl (2 h)	APAP (1 h)	APAP (2 h)
Fasted glucose (mmol/mL)	3.68 ± 0.32	3.96 ± 0.25	4.27 ± 0.34
Serum insulin (mIU/L)	17.59 ± 2.80	16.75 ± 1.62	15.23 ± 2.30
Serum glucagon (nmol/L)	121.47 ± 14.82	106.94 ± 5.27	104.12 ± 5.35
HDL-C (mmol/L)	22.05 ± 2.71	16.85 ± 3.09	28.17 ± 6.8
LDL-C (mmol/L)	0.38 ± 0.04	0.51 ± 0.09	0.47 ± 0.03
ALT (U/L)	52.87 ± 3.84	61.94 ± 7.85	69.35 ± 8.23
AST (U/L)	150.75 ± 15.94	175.72 ± 9.06	186.08 ± 10.21
Blood-APAP (μg/ml)	-	114.85 ± 12.09	92.49 ± 11.22
Serum-FGF21 (pg/mL)	603.33 ± 57.95	448.33 ± 43.68	431.67 ± 35.12^a^
Liver-FGF21 (ng/mg)	583.26 ± 45.25	457.89 ± 31.69	417.62 ± 28.77^a^

Note:

Ctrl, control; APAP, acetaminophen; HDL-C, high-density lipoprotein cholesterol; LDL-C, low-density lipoprotein cholesterol; ALT, glutamic-pyruvic transaminase; AST, glutamic-oxaloacetic transaminase; FGF21, fibroblast growth factor 21.

^a^*P* < 0.05 vs Ctrl statistically analyzed through Student's *t* test.

### The immediate effect of APAP on hepatocellular FGF21 expression in mice

**Figure 2:**
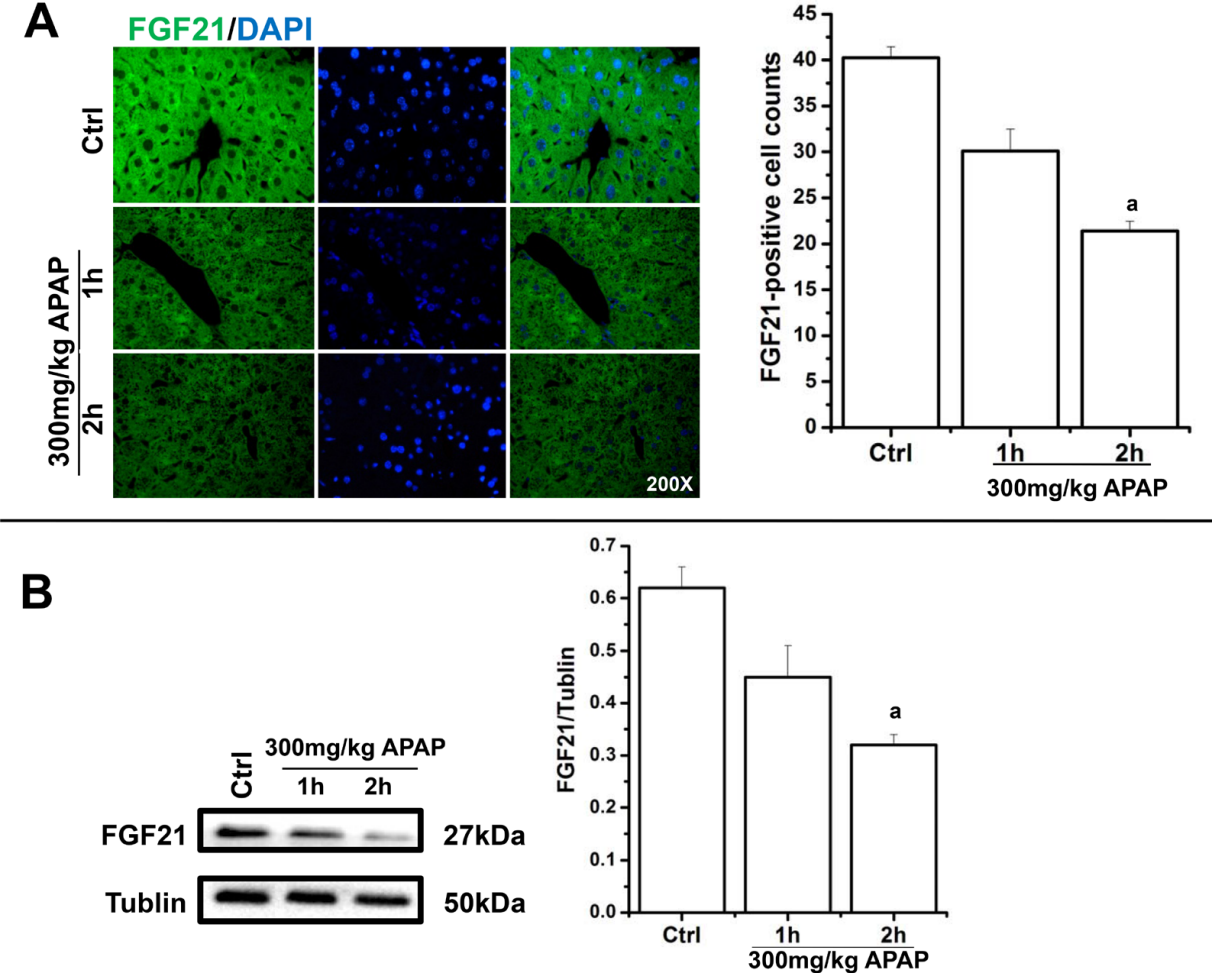
The negative regulation of APAP on FGF21 levels in liver cells APAP-exposed livers showed reduced FGF21-positive cell numbers time-dependently, as revealed in immunofluorescencence assays (magnification 400×). Additionally, quantitative immunoblotting data indicated that APAP-exposed livers exhibited downregulated FGF21 expression in a time-dependent manner. Statistical results were analyzed using one-way ANOVA followed by Student's *t* test. Final data were expressed as mean ± SD. Note: vs. Ctrl (control), ^a^*P* < 0.05.

To further assay the immunophenotype of FGF21 in APAP-treated livers, an immunofluorescencence test was conducted. In cytohistologic observation, APAP-exposed livers showed downregulated FGF21-positive cell counts in a time-dependent manner, in which the number of cells expressing FGF21 in the cytoplasm was lower than that in APAP-free livers, especially during the 2-h exposure (*P* < 0.05) (Figure 2A). As revealed in quantitative analysis of FGF21 expression, immunoblotting data found that APAP-exposed livers displayed reduced FGF21 levels in the cells, in which 2 h APAP treatment had statistical significance in comparison to that in unexposed livers (*P* < 0.05) (Figure 2B).

## DISCUSSION

APAP, a widely used antipyretic and analgesic medication, has adverse effects when taken in overdose, such as time- and dose-dependent liver damage. APAP is commonly associated with time-dependent liver impairment, even at the recommended dose. Hepatotoxicity, induced by a quinone metabolite that is toxic to liver cells, such as N-acetyl-p-benzoquinonimine (NAPQI) [9–11], is one of the main side effects of APAP. However, early liver impairment induced by APAP is difficult to clinically diagnose. Therefore, alternative biomarkers with apparent sensitivity should be explored in early APAP liver impairment. In the current study, human serological data suggested that APAP-treated patients showed comparable levels of FGF21 in the group with no significant liver impairment, implying the potential application of FGF21 in monitoring early liver impairment induced by APAP. Further, more detailed observations was investigated through the animal study.

In physiology, the liver is an important organ capable of digestion, hormone production, and detoxification, and its high-volume biochemical reactions are indispensable for normal bodily functions [12–13]. On the basis of hepatocellular bioactivity, the metabolic ability can be reflected in detoxification of clinical drugs, including APAP. Once superthreshold use is reached, APAP can damage liver cells by a group of biological reactions, such as inflammation, oxidative stress, and cell necrosis [14–15]. All these hepatocellular events can be identified through biochemical diagnosis. However, early liver impairment induced by APAP should also be investigated. PARP is linked to the fundamental excision repair pathway, mainly through catalyzing the poly(ADP-ribosyl) role of stabilizing chromatin architecture and DNA metabolism. The modification of DNA-dependent way is found in connection with modulating differentiation, proliferation, cell survival [16–17]. FGF21, an endogenous hormone produced from liver cells, can exert cell reparation against cytotoxicity [18] as well as contributing to the maintenance of metabolism homeostasis through the hormonal pathway [19]. On the basis of the underlying biofunction of FGF21, we propose that FGF21 expression may be useful for screening APAP-induced early liver impairment through the hormonal pathway. In this study, clinical and rodent experiment findings indicated that the FGF21-impaired expression induced by APAP was more significant than those parameters referenced in clinical diagnosis. APAP-treated patients exhibited lower FGF21 levels in plasma time-dependently, while liver functions was validated by clinical biochemical assays. In the APAP-exposed mice models (1- or 2-h exposure), FGF21 levels in sera and liver cells were reduced in early liver impairment, as revealed by serological examination and immunoassay. On the basis of the current preliminary data, we propose that FGF21 functions as an attractive biomarker in reflecting liver impairment induced by APAP, even in early stages.

## MATERIALS AND METHODS

### Clinical study

All participants aged 20 to 35 years old with detectable diseases were excluded. In particular, the recruited patients who had liver diseases (such as hepatitis or fatty liver) were excluded on the basis of oral consultation and biochemical assay. These patients experienced a slight fever before taking APAP. Blood samples of all subjects were collected from the elbow vein during 1- and 2-hour time points. The plasma was harvested immediately and then stored at −20°C for further serological diagnosis using an automatic blood analyzer. All subjects signed informed consent forms from the Department of Physical Examination before sera collection. Additionally, all the clinical procedures were conducted according to the Ethical Guidelines of the Declaration of Helsinki [20].

### Instrumental analysis

Human plasma and mice serum samples were isolated according to referenced biological methods [21]. After deproteinization, purified APAP standard (Yuanye Biology Co. Ltd. Shanghai, China) was used as an internal control. High-performance liquid chromatography (HPLC; LC-20A, Shimadzu, Japan) was performed using an SIL-20AC detector and ASTON RG C18 column (4.6 mm × 50 mm i.d., 5 μm; China). The ratio of acetonitrile:isopropanol:purified water in 23:8:69 in triethylamine solution (final pH=6.9) was used as a mobile phase at the flow speed of 1.1 ml/min. The APAP concentration was normalized and calculated as μg/ml.

### Medication/reagents

Commercially available acetaminophen (APAP) was purchased from Sinopharm Group Guangdong Medi-World Pharmaceutical Co., Ltd. (Foshan, China). Other necessary materials are indicated in follow-up experimental sections.

### Animal study

Adult male Kunming (KM) mice, aged 7 weeks old, were obtained from the Laboratory Animal Center of Guilin Medical University. This animal study was approved by the Institutional Ethical Committee of Guilin Medical University.

All mice were housed in an animal room with controlled temperature (24 ± 2°C) and cycled light (12 h light/12 h dark). After acclimatization for one week, the mice in the APAP group were treated with a single dose of APAP solution in saline buffer (300 mg/kg, w/w) by oral gavage, while the control mice were administered with an equivalent volume of saline buffer. After fasting for 1 and 2 hours, all mice were killed through cervical dislocation, and serum was collected and immediately stored. A liver specimen was also harvested for subsequent biological assays.

### Biochemical analyses and routine stain

A group of liver functional enzymes was analyzed using an automatic biochemical analyzer after the collection of plasma or serum.

Fasting serum human-derived FGF1 and mouse-derived FGF1, insulin, glucagon, triglycerides, and free fatty acid were measured through commercially available enzyme-linked immunosorbent assay (ELISA) kits (Shanghai Elisa Biotech Co., Ltd., China).

Cytohistologically, mouse liver tissue was fixed with 10% neutral formalin (w/v) and prepared as paraffin-embedded block. Five-micrometer sections were subjected to hematoxylin and eosin (HE) staining (Boster, Wuhan, China).

### Immunofluorescencent staining

In this procedure, formalin-fixed, paraffin-embedded mouse livers were incubated with 5% bovine serum albumin (BSA) in 0.05% phosphate-buffered saline (PBS)-Tween for 1 h to permeabilize hepatic cells and block non-specific protein-protein interactions, and was then stained with rabbit-anti FGF21 primary antibody (1:200, Abcam, UK) overnight at 4°C. The secondary antibody (green) was used with DyLight 488 goat anti-rabbit IgG (H+L) buffer (1:250, Abcam, UK) for 1 h. 4′,6-Diamidino-2-phenylindole (DAPI) was used to stain the cell nuclei (blue) (Abcam, UK) before imaging and cell counting [22].

### Western blot assay

Hepatic lysate was isolated using radioimmunoprecipitation assay (RIPA) buffer supplemented 1 mM phenylmethylsulfonyl fluoride (PMSF) protein inhibitor (Beyotime Biotechnology, China). Forty micrograms of protein in each lane was electrophoresed with 10% sodium dodecyl sulfate-polyacrylamide gel electrophoresis (SDS-PAGE) and then transferred to 0.2-μm polyvinyldene fluoride (PVDF) membranes (Millipore, MA, USA). After blocking with 5% non-fat milk solution (Yili Industrial Group Co., Ltd, China) for 1 h, the membrane was incubated with diluted rabbit-anti-FGF21 primary antibody (1:1000, Abcam, UK), rabbit-anti-PARP, and cleaved PARP (1:1000, Cell Signaling Technology, USA) overnight at 4°C, developed using a horseradish peroxidase (HRP)-coupled secondary antibody (Beyotime Biotechnology) for 1 h. After adding the chemiluminescent reagent, the membrane was imaged and captured using a gel imager (BIO-RAD, USA). Internal α-tubulin protein (Beyotime Biotechnology) was used as a housekeeping control for target protein normalization [23–24].

### Statistical analysis

Statistical results were generated using the Statistical Product and Service Solutions (SPSS) 19.0 software. Differences between compared groups were analyzed through a one-way analysis of variance (ANOVA) test followed by Student's *t* test. Final results were indicated as mean ± SD. A *p* < 0.05 was considered statistically significant.

## CONCLUSIONS

Collectively, our preliminary evidence demonstrates that abnormal FGF21 expression can be detected in early APAP-induced liver impairment. These comparable data highlighted that FGF21 may be a biosensitive indicator of APAP-impaired liver.
